# Supplementary material for: The HIV-1 latent reservoir is largely sensitive to circulating T cells
Source: eLife. 2020 Oct 6;9:e57246. doi: 10.7554/eLife.57246 (PMC7593086; doi:10.7554/eLife.57246)
Supplement: Supplementary file 7. [file elife-57246-supp7.docx]

**Supplementary File 7:** Correlation between T cell targeting (breadth and magnitude) and the size of the replication-competent reservoir as measured by infectious units per million (IUPM)

| Variable 1 | | Variable 2 | Statistical Test | r | p-value | Number pairs |
| --- | --- | --- | --- | --- | --- | --- |
| T cell breadth | Summed magnitude of T cell response | | Spearman Rank | r=0.57 | p=0.003 | 25 pairs |
|  | Size of protein (1000DA) | | Spearman Rank | r=0.67 | p=0.055 | 9 pairs |
|  | ART initiated during acute versus chronic HIV-1 infection | | Two—tailed Mann Whitney Test |  | p=0.792 |  |
|  | Gender | | Two—tailed Mann Whitney Test |  | p>0.999 |  |
|  | IUPM | | Spearman Rank, | r=0.047 | p=0.822 | 25 pairs |
| Summed magnitude of T cell response | ART initiated during acute versus chronic HIV-1 infection | | Two—tailed Mann Whitney Test |  | p=0.331 |  |
|  | Gender | | Two—tailed Mann Whitney Test |  | p=0.487 |  |
|  | IUPM | | Spearman Rank | r=0.21 |  | 25 pairs |
| IUPM | Gender | | Mann-Whitney Test |  | p=0.192 |  |
|  | Length of durable suppression | | Spearman Rank | r=0.098 | p=0.642 | 25 pairs |
