# Supplementary material for: The HIV-1 latent reservoir is largely sensitive to circulating T cells
Source: eLife. 2020 Oct 6;9:e57246. doi: 10.7554/eLife.57246 (PMC7593086; doi:10.7554/eLife.57246)
Supplement: Supplementary file 8. — Correlation between T cell breadth and summed magnitude of T cell response to HIV-1 protein in PLWH on ART (n = 25 participants, n = 166 epitopes), and adjusted for escape variants (n = 23 participants, n = 102 epitopes excluding 49 epitopes at which escape was observed), measured by IFN-γ ELISpot and the size of the replication-competent reservoir as measured by infectious units per million (IUPM) using Spearman Rank. [file elife-57246-supp8.docx]

**Supplementary File 8:** Correlation between T cell breadth and summed magnitude of T cell response to HIV-1 protein in PLWH on ART (n=25 participants, n=166 epitopes), and adjusted for escape variants (n=23 participants, n=102 epitopes excluding 49 epitopes at which escape was observed), measured by IFN-y ELISpot and the size of the replication-competent reservoir as measured by infectious units per million (IUPM) using Spearman Rank.

| **Total (n=166 epitopes)** | | | | | |
| --- | --- | --- | --- | --- | --- |
|  | **IUPM versus breadth** | | **IUPM versus summed magnitude** | | |
| **Protein** | **r** | **p-value** | **r** | **p-value** | **Number of pairs** |
| Gag | -0.252 | 0.297 | -0.018 | 0.940 | 19 |
| Pol | 0.564 | 0.015 | 0.476 | 0.046 | 18 |
| Env | -0.252 | 0.428 | 0.056 | 0.869 | 12 |
| Nef | 0.215 | 0.439 | 0.304 | 0.271 | 15 |
| Vif | 0.000 | >0.999 | 0.500 | >0.999 | 3 |
| Vpr | 0.082 | >0.999 | 0.095 | 0.840 | 8 |
| Vpu | N/A | -^A^ | N/A | - | 1 |
| Tat | -0.775 | 0.500 | -0.800 | 0.333 | 4 |
| Rev | N/A | - | N/A | - | 2 |
|  |  |  |  |  |  |
| **Adjusted for Escape (n=102 epitopes)** | | | | | |
|  | **IUPM versus breadth** | | **IUPM versus summed magnitude** | | |
| **Protein** | **r** | **p-value** | **r** | **p-value** | **Number of pairs** |
| Gag | -0.218 | 0.451 | -0.189 | 0.522 | 14 |
| Pol | 0.601 | 0.016 | 0.459 | 0.076 | 16 |
| Env | 0.000 | 0.451 | -0.100 | 0.950 | 5 |
| Nef | 0.519 | 0.089 | 0.378 | 0.228 | 12 |
| Vif | N/A | - | N/A | - | 2 |
| Vpr | 0.131 | >0.999 | -0.100 | 0.950 | 5 |
| Vpu | N/A | - | N/A | - | 0 |
| Tat | N/A | - | N/A | - | 2 |
| Rev | N/A | - | N/A | - | 1 |

^A^ – insufficient pairs for analysis
