# Supplementary material for: The HIV-1 latent reservoir is largely sensitive to circulating T cells
Source: eLife. 2020 Oct 6;9:e57246. doi: 10.7554/eLife.57246 (PMC7593086; doi:10.7554/eLife.57246)
Supplement: Supplementary file 9. — Escape in the HIV-1 reservoir is consistently lower in T cell epitopes that fall within conserved immunogen vaccines than mapped epitopes that fall outside of the immunogens. [file elife-57246-supp9.docx]

**Supplementary File 9:** Reactive T cell epitopes in PLWH on ART (n=23 participants, 151 *total* mapped epitopes including 49 *escape* epitopes) are targeted by conserved immunogen vaccines. Escape in the HIV-1 reservoir is consistently lower in T cell epitopes that fall within conserved immunogen vaccines than mapped epitopes that fall outside of the immunogens.

|  |  |  |  | Total # epitopes across cohort (n=151) | | | | # epitopes/participant | |
| --- | --- | --- | --- | --- | --- | --- | --- | --- | --- |
| Immunogen | Size | Protein regions | Clade(s) | In/Out of vaccine | Escape/ Total^A^ | % Escape | p-value^B^ | Ave. total (range) | Ave. escape (range) |
| **HIVconsv** ([79](#_ENREF_79)) | 778aa | Gag, Pol, Vif, Env | Multiclade^C^ | In | 5/41 | 12.2% | p=0.002 | 2 (0-5) | 0 (0-1) |
|  |  |  |  | Out | 44/110 | 40.0% |  | 5 (0-16) | 2 (0-7) |
| **tHIVconsvX** ([49](#_ENREF_49)) | 872aa | Gag, Pol | 2 mosaics^D^ | In | 9/59 | 15.3% | p=0.001 | 3 (0-6) | 0 (0-2) |
|  |  |  |  | Out | 40/92 | 43.5% |  | 4 (0-16) | 2 (0-7) |
| **HTI** ([80](#_ENREF_80)) | 529aa | Gag, Pol, Vif, Nef | B | In | 8/28 | 28.6% | p=0.793 | 1 (0-3) | 0 (0-2) |
|  |  |  |  | Out | 41/123 | 33.3% |  | 5 (0-17) | 2 (0-7) |
| **p24 CE** ([81](#_ENREF_81)) | 124aa | Gag | 2 mosaics^D^ | In | 3/16 | 18.8% | p=0.340 | 1 (0-4) | 0 (0-1) |
|  |  |  |  | Out | 46/135 | 34.1% |  | 6 (1-18) | 2 (0-7) |

^A^ escape/total: For each immunogen the sum of numerators = 49 epitopes in which escape was confirmed and sum of denominators = 151 reactive T cell epitopes mapped in 23 PLWH on ART

^B^ Pearson’s χ^2^ test with Yates correction, significant at p<0.05, to compare the distribution of mapped T cell epitopes that fall within conserved immunogen vaccines (with a minimal epitope length of 9aa based on HXB2 location) and that fall outside conserved immunogen vaccines.

^c^ Multiclade: multicomponent vaccine encoding antigens from various clades of HIV-1

^D^ Mosaics: complementary sequences, that in combination, provide boarder coverage of potential T cell epitopes
