# Supplementary material for: The HIV-1 latent reservoir is largely sensitive to circulating T cells
Source: eLife. 2020 Oct 6;9:e57246. doi: 10.7554/eLife.57246 (PMC7593086; doi:10.7554/eLife.57246)
Supplement: Supplementary file 10. [file elife-57246-supp10.docx]

**Supplementary File 10:** Comparison of Approaches to Assess Pre-ART Escape in the Latent HIV-1 Reservoir

|  | Deng et al. ([42](#_ENREF_42)) | Warren et al. | |
| --- | --- | --- | --- |
| # participants (acute:chronic) | 25 (10:15) | 25 (4:21) | |
| HIV-1 proteome examined | Gag | Whole proteome | |
| Latent reservoir sequencing | Proviral (n= 25);  Replication-competent (n=7) | Replication-competent (n=23) | |
| Sequencing method (# participants) | Deep sequencing: Pac Bio and Miseq (25) | Near full-length: Pac Bio (22) | Deep sequencing of epitope: Primer ID (1)^A^ |
| Mean sequencing depth (range) | >1000 | 25 (1-71) | 3,700 |
| Total Epitopes with sequencing data | 169 | 151 | |
| Variant differs from Clade B | 72 (43%) | 115 (76%) | |
| Variant *does not* differ from Clade B | 97 (57%) | 36 (26%) | |
|  |  |  | Limited to Gag epitopes examined in Deng et. al. ^B^ |
| # of epitopes empirically tested | 36  (a subset of total) | 151 | 33 |
| Escape | 34 | 49 | 7 |
| No escape | 2 | 102 | 26 |
| Escape frequency | 94%  (34/36) | 32% (49/151) | 21%  (7/33) |
| Average within-epitope escape frequency^A^ | 98% | 27% | 22% |
| Definition of Escape (as measured by ex vivo IFN-γ ELISpot) | No T cell response, or *partial* decreased | ≥50% decrease in the T cell magnitude | |

^A^ Partial HIV gene DNA sequencing for two participants as shown in Supp Figure 4

^B^ Within-epitope escape frequency limited to individuals treated during chronic infection (compared to Deng et al.)
