# Supplementary material for: The HIV-1 latent reservoir is largely sensitive to circulating T cells
Source: eLife. 2020 Oct 6;9:e57246. doi: 10.7554/eLife.57246 (PMC7593086; doi:10.7554/eLife.57246)
Supplement: Transparent reporting form [file elife-57246-transrepform.docx]

***eLife’s* transparent reporting form**

We encourage authors to provide detailed information *within their submission* to facilitate the interpretation and replication of experiments. Authors can upload supporting documentation to indicate the use of appropriate reporting guidelines for health-related research (see [EQUATOR Network](http://www.equator-network.org/%20)), life science research (see the [BioSharing Information Resource](https://biosharing.org/)), or the [ARRIVE guidelines](http://www.plosbiology.org/article/info:doi/10.1371/journal.pbio.1000412) for reporting work involving animal research. Where applicable, authors should refer to any relevant reporting standards documents in this form.

If you have any questions, please consult our Journal Policies and/or contact us: [editorial@elifesciences.org](mailto:editorial@elifesciences.org).

**Sample-size estimation**

- You should state whether an appropriate sample size was computed when the study was being designed
- You should state the statistical method of sample size computation and any required assumptions
- If no explicit power analysis was used, you should describe how you decided what sample (replicate) size (number) to use

Please outline where this information can be found within the submission (e.g., sections or figure legends), or explain why this information doesn’t apply to your submission:

The is a descriptive analysis of HIV specific T cell responses and viral escape in people living with HIV (PLWH) on antiretroviral therapy (ART). This was not a hypothesis testing study and therefore no power calculations were performed a priori.

Note, the level detail in this study, proteome-wide mapping, quantitative viral outgrowth assay, fully length sequencing of reservoir virus and comprehensive testing of viral variants has not before been previously attempted. Examples of related studies is the 2003 paper by Addo et al who performed proteome-wide sequencing of PLWH on ART in 12 individuals without any measurements of the reservoir or assessment of escape. In 2015, Deng et al performed sequencing of total HIV reservoir (not replication competent, therefore no IUPM calculations) in 25 PLWH on ART, then mostly used bioinformatics to predict T cell epitopes and virus escape. Empirical studies examining T cell responses and escape were performed on selected epitopes in no more than half of all subjects. To our knowledge, this study is the most detailed and comprehensive of its kind in PLWH on ART.

**Replicates**

- You should report how often each experiment was performed
- You should include a definition of biological versus technical replication
- The data obtained should be provided and sufficient information should be provided to indicate the number of independent biological and/or technical replicates
- If you encountered any outliers, you should describe how these were handled
- Criteria for exclusion/inclusion of data should be clearly stated
- High-throughput sequence data should be uploaded before submission, with a private link for reviewers provided (these are available from both GEO and ArrayExpress)

Please outline where this information can be found within the submission (e.g., sections or figure legends), or explain why this information doesn’t apply to your submission:

All information regarding replicates, pass-fail criteria, data exclusion are described throughout the Result section, with more details in the Methods section.

Outgrowth viral sequences have been submitted to GenBank and Sequence Read Archive (SRA) and are awaiting accession numbers.

**Statistical reporting**

- Statistical analysis methods should be described and justified
- Raw data should be presented in figures whenever informative to do so (typically when N per group is less than 10)
- For each experiment, you should identify the statistical tests used, exact values of N, definitions of center, methods of multiple test correction, and dispersion and precision measures (e.g., mean, median, SD, SEM, confidence intervals; and, for the major substantive results, a measure of effect size (e.g., Pearson's r, Cohen's d)
- Report exact p-values wherever possible alongside the summary statistics and 95% confidence intervals. These should be reported for all key questions and not only when the p-value is less than 0.05.

Please outline where this information can be found within the submission (e.g., sections or figure legends), or explain why this information doesn’t apply to your submission:

Statistical analysis methods are described throughout the Result section and accompanying figure legends, including in supplemental materials.

T cell data (magnitude, breadth) are mostly provided as raw data in tables in the primary manuscript with all detailed (HXB2 location, sequence, HLA-restriction) data listings in supplemental files.

Statistical tests are listed for each experiment with mean, median and range listed as appropriate. Again, raw data are provided in supplemental files.

Exact p-values are reported throughout the paper.

(For large datasets, or papers with a very large number of statistical tests, you may upload a single table file with tests, Ns, etc., with reference to sections in the manuscript.)

**Group allocation**

- Indicate how samples were allocated into experimental groups (in the case of clinical studies, please specify allocation to treatment method); if randomization was used, please also state if restricted randomization was applied
- Indicate if masking was used during group allocation, data collection and/or data analysis

Please outline where this information can be found within the submission (e.g., sections or figure legends), or explain why this information doesn’t apply to your submission:

Group allocation does not apply to this submission. This was not intervention study and participants were not allocated/randomized to any group.

**Additional data files (“source data”)**

- We encourage you to upload relevant additional data files, such as numerical data that are represented as a graph in a figure, or as a summary table
- Where provided, these should be in the most useful format, and they can be uploaded as “Source data” files linked to a main figure or table
- Include model definition files including the full list of parameters used
- Include code used for data analysis (e.g., R, MatLab)
- Avoid stating that data files are “available upon request”

Please indicate the figures or tables for which source data files have been provided:

Source/raw data has been provided for Table 2 (Supplementary files 5 and 6 ) and Figure 4 (Supplemental Files 4,5,6), Figure 3 – figure supplement 4 (Supplemental File 1), Figure 4 – figure supplement 2 (Supplemental Files 5 and 6) and Supplementary files 7 and 8 (Supplemental Files and 6).
